# Increased Hospitalizations Involving Fungal Infections during COVID-19 Pandemic, United States, January 2020–December 2021

**DOI:** 10.3201/eid2907.221771

**Published:** 2023-07

**Authors:** Jeremy A.W. Gold, Stacey Adjei, Adi V. Gundlapalli, Ya-Lin A. Huang, Tom Chiller, Kaitlin Benedict, Mitsuru Toda

**Affiliations:** Centers for Disease Control and Prevention, Atlanta, Georgia, USA

**Keywords:** fungi, invasive fungal infections, mycoses, hospitalizations, aspergillosis, candidiasis, blastomycosis, coccidioidomycosis, cryptococcosis, histoplasmosis, mucormycosis, pneumonia, *Pneumocystis*, international classification of diseases, COVID-19, respiratory infections, severe acute respiratory syndrome coronavirus 2, SARS-CoV-2, SARS, coronavirus disease, zoonoses, viruses, coronavirus, United States

## Abstract

Hospitalizations involving fungal infections increased 8.5% each year in the United States during 2019–2021. During 2020–2021, patients hospitalized with COVID-19–associated fungal infections had higher (48.5%) in-hospital mortality rates than those with non–COVID-19–associated fungal infections (12.3%). Improved fungal disease surveillance is needed, particularly during respiratory virus pandemics.

In the United States, fungal infections impose considerable healthcare costs (≈$6.7 billion during 2018) and cause substantial illness and death (>7,000 deaths during 2021) (*1*,*2*). Fungi causing serious infections include yeasts, such as *Candida*, *Cryptococcus*, and *Pneumocystis* spp.; molds, such as *Aspergillus* spp. and Mucorales; and dimorphic fungi, such as *Blastomycoses*, *Coccidioides*, and *Histoplasma* spp. (*2*). Risk factors vary but generally are associated with environmental exposures; underlying immunocompromising conditions, such as solid organ or stem cell transplantation, cancer, and immunosuppressive medications; and critical illness (*2*–*4*).

COVID-19 infection is a substantial risk factor for certain fungal infections, particularly those caused by invasive molds, likely because of COVID-19–related immune system dysregulation and immunosuppressive therapies, such as corticosteroids or other immunomodulatory medications (*3*). US vital statistics data showed that deaths from fungal infections increased during the COVID-19 pandemic (*2*). However, additional data on fungal infections during the pandemic, including hospitalization rates and healthcare utilization, are lacking because many fungal diseases are not reportable in the United States (https://www.cdc.gov/fungal/fungal-disease-reporting-table.html). Those data might help inform public health planning and clinical practice. Therefore, we analyzed a large healthcare services database to determine rates, patient demographic features, and healthcare utilization for fungal infection–related hospitalizations during the COVID-19 pandemic.

## The Study

The Premier Healthcare Database, Special COVID-19 Release (PHD-SR), is a US, hospital-based, all-payer database used by the Centers for Disease Control and Prevention to inform COVID-19 response activities (*5*,*6*). The database contains deidentified records from >1,000 nongovernment, community, and teaching hospitals that contributed inpatient data during the analytic period. We used diagnosis codes from the International Classification of Diseases, 10th Revision, Clinical Modification (ICD-10-CM), listed for each hospitalization and identified hospitalizations involving fungal infections (fungal hospitalizations) and COVID-19 (COVID-19 hospitalizations) during January 1, 2019–December 31, 2021 (Appendix Table 1). We defined COVID-19–associated fungal hospitalizations as those in which both a COVID-19 and fungal infection diagnosis were listed during the same hospitalization.

We estimated annual hospitalization rates (per 10,000 population) by fungal infection type and calculated average annual percentage change during 2019–2021. For COVID-19–associated fungal hospitalizations (2020–2021 only), we calculated hospitalization rates per 10,000 COVID-19 hospitalizations. We stratified 2020–2021 fungal hospitalizations by COVID-19 association and fungal infection type and compared patient demographics, US hospital census regions and urban–rural classifications (https://www.cdc.gov/nchs/data_access/urban_rural.htm), lengths of hospital stays, intensive care unit (ICU) admissions, invasive mechanical ventilation (IMV) receipt, and in-hospital deaths. We assessed annual trends in fungal hospitalizations by using Cochran-Armitage tests and compared fungal hospitalizations according to COVID-19 status by using χ^2^ tests.

During 2019–2021, a total of 59,212 fungal hospitalizations were identified in the PHD-SR. Rates of fungal hospitalizations (per 10,000 hospitalizations) increased from 22.3 in 2019 to 25.0 in 2020 and 26.8 in 2021 (p<0.01), representing an average annual percentage change of 8.5% (Table 1). Average annual rates of hospitalization significantly increased for each fungal infection, except for those caused by *Pneumocystis* spp., *Cryptococcus* spp., and other specified fungi (Table 1).

**Table 1:** Hospitalization rates for invasive fungal infections associated with COVID-19 in study of increased hospitalizations involving fungal infections during COVID-19 pandemic, United States, January 2020–December 2021*

Fungal pathogen	All fungal hospitalizations, n = 59,212					COVID-19–associated hospitalizations,† n = 5,288			
	2019	2020	2021	p value‡	% Change§	2020	2021	p value‡	% Change¶
Pathogenic fungi	22.3	25.0	26.8	<0.01	8.5	43.1	57.4	<0.01	24.9
*Candida*	4.2	5.3	5.6	<0.01	12.4	11.2	10.9	0.69	−2.4
*Aspergillus*	4.3	4.2	5.3	<0.01	9.4	7.9	18.9	<0.01	58.2
*Coccidioides*	3.2	4.0	4.3	<0.01	13.0	6.6	7.4	0.15	10.4
*Pneumocystis*	2.6	2.4	2.5	0.08	−2.7	1.9	2.6	0.03	25.4
*Histoplasma*	1.4	1.6	1.6	<0.01	5.6	1.1	1.6	0.03	32.1
*Cryptococcus*	1.3	1.4	1.2	0.46	−2.0	1.2	1.4	0.48	11.6
*Blastomyces*	0.3	0.3	0.4	<0.01	11.7	0.2	0.5	<0.01	65.6
Mucorales species	0.3	0.3	0.4	<0.01	17.9	0.7	1.1	0.02	39.8
Other specified fungi	1.5	1.6	1.5	0.38	−1.9	1.7	2.5	<0.01	32.9
Unspecified	4.0	4.9	5.1	<0.01	10.8	12.2	12.7	0.47	4.1

*Rates are per 10,000 hospitalizations. The total number of hospitalizations per year was 8,884,472 in 2019, 7,640,470 in 2020 (424,475 COVID-19–associated), and 7,559,882 in 2021 (601,831 COVID-19–associated). Patients could have >1 fungal infection in a given hospitalization, which occurred for <5% of hospitalizations. †Hospitalizations of patients with COVID-19–associated fungal infections. ‡Calculated by using Cochran-Armitage tests. §Average annual percentage change during 2019–2021. ¶Annual percentage change during 2020–2021.

During 2020–2021, a total of 5,288 (13.4%) of 39,423 fungal hospitalizations were COVID-19–associated. Rates of COVID-19–associated fungal hospitalizations (per 10,000 COVID-19 hospitalizations) increased by 24.9% (43.1% to 57.4%; p<0.01). Annual rates increased significantly for COVID-19–associated fungal hospitalizations involving blastomycosis (0.2 to 0.5 [65.6% change]; p<0.01), aspergillosis (7.9 to 18.9 [58.2% change]; p<0.01), mucormycosis (0.7 to 1.1 [39.8% change]; p = 0.02), histoplasmosis (1.1 to 1.6 [32.1% change]; p = 0.03), pneumocystosis (1.9 to 2.6 [25.4% change]; p = 0.03), and other specified mycoses (1.7 to 2.5 [32.9% change]; p<0.01). Compared with non–COVID-19–associated fungal hospitalizations, COVID-19–associated fungal hospitalizations more frequently involved aspergillosis (27.8% vs. 16.9%; p<0.01), mucormycosis (1.8% vs. 1.4%; p = 0.03), and unspecified mycoses (24.3% vs. 18.5%; p<0.01) and, in general, less frequently involved other fungal infection types (Table 2).

**Table 2:** Hospitalizations for fungal infections according to COVID-19 status in study of increased hospitalizations involving fungal infections during COVID-19 pandemic, United States, January 2020–December 2021*

Fungal pathogen	Any fungal infection	COVID-19–associated	Non–COVID-19–associated	p value
Total no.	39,423	5,288	34,135	NA
*Candida*	8,289 (21.0)	1,135 (21.5)	7,154 (21.0)	0.40
*Aspergillus*	7,248 (18.4)	1,471 (27.8)	5,777 (16.9)	<0.01
*Coccidioides*	6,278 (15.9)	723 (13.7)	5,555 (16.3)	<0.01
*Pneumocystis*	3,718 (9.4)	235 (4.4)	3,483 (10.2)	<0.01
*Histoplasma*	2,386 (6.1)	139 (2.6)	2,247 (6.6)	<0.01
*Cryptococcus*	2,022 (5.1)	138 (2.6)	1,884 (5.5)	<0.01
*Blastomyces*	543 (1.4)	41 (0.8)	502 (1.5)	<0.01
Mucorales species	569 (1.4)	94 (1.8)	475 (1.4)	0.03
Other specified fungi	2,300 (5.8)	221 (4.2)	2,079 (6.1)	<0.01
Unspecified fungi	7,599 (19.3)	1,286 (24.3)	6,313 (18.5)	<0.01

*Values are no. (%) hospitalizations except as indicated. Patients could have >1 fungal infection in a given hospitalization, which occurred for <5% of hospitalizations. p values were calculated by using χ^2^ tests comparing COVID-19–associated and non–COVID-19–associated fungal hospitalizations. NA, not applicable.

Median patient age was 63 (interquartile range [IQR] 52–72) years for COVID-19–associated hospitalizations versus 61 (IQR 46–72) years for non–COVID-19–associated hospitalizations (p<0.01) (Figure). Compared with hospitalizations of patients with non–COVID-19–associated fungal infections, hospitalizations of patients with COVID-19–associated fungal infections more frequently involved those who were male (59.9% vs. 57.5%; p<0.01) and Hispanic/Latino (18.8% vs. 11.7%; p<0.01); occurred in hospitals located in the western United States (29.1% vs. 27.5%; p<0.01); involved longer hospital stays (21 [IQR 11–35] days vs. 9 [IQR 4–17] days; p<0.01); and involved ICU-level care (70.0% vs. 35.5%; p<0.01), IMV receipt (64.4% vs. 22.5%; p<0.01), and increased in-hospital deaths (48.5% vs. 12.3%; p<0.01) (Table 3).

**Figure:**
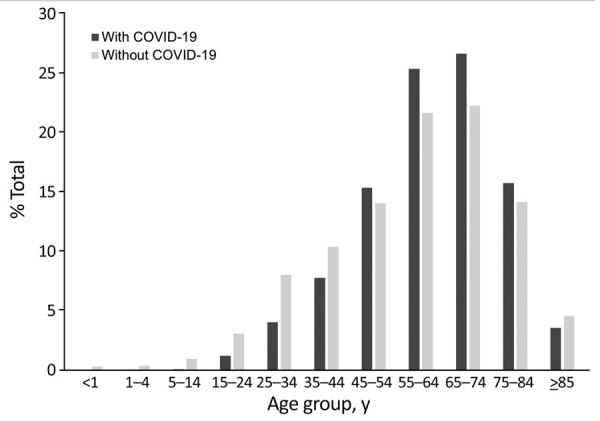
Age distribution of patients in study of increased hospitalizations involving fungal infections during COVID-19 pandemic, United States, January 2020–December 2021. Hospitalizations for fungal infections were COVID-19–associated (n = 5,288) or non–COVID-19–associated (n = 34,135).

**Table 3:** Demographic features and outcomes of patients with fungal infections by COVID-19 status in study of increased hospitalizations involving fungal infections during COVID-19 pandemic, United States, January 2020–December 2021*

Characteristics	Any fungal infection	COVID-19–associated	Non–COVID-19–associated	p value†
Total no. hospitalizations	39,423	5,288	34,135	NA
Median age, y (range)	61 (47–72)	63 (52–72)	61 (46–72)	<0.01
Sex‡				
M	22,779 (57.8)	3,166 (59.9)	19,613 (57.5)	<0.01
F	16,634 (42.2)	2,121 (40.1)	14,513 (42.5)	<0.01
Race/ethnicity				
White, NH	18,359 (46.6)	2,131 (40.3)	16,228 (47.5)	<0.01
Black, NH	5,907 (15.0)	771 (14.6)	5,136 (15.0)	0.38
Hispanic or Latino	5,002 (12.7)	993 (18.8)	4,009 (11.7)	<0.01
Asian, NH	878 (2.2)	106 (2.0)	772 (2.3)	0.24
Other, NH	1,331 (3.4)	210 (4.0)	1,121 (3.3)	0.01
Unknown	7,946 (20.2)	1,077 (20.4)	6,869 (20.1)	0.68
US census region				
South	16,241 (41.2)	2,141 (40.5)	14,100 (41.3)	0.26
West	10,914 (27.7)	1,538 (29.1)	9,376 (27.5)	0.01
Midwest	7,385 (18.7)	943 (17.8)	6,442 (18.9)	0.07
Northeast	4,883 (12.4)	666 (12.6)	4,217 (12.4)	0.62
Urban location	35,938 (91.2)	4,801 (90.8)	31,137 (91.2)	0.31
Healthcare utilization and outcomes				
Median length of stay, d (range)	10 (5–20)	21 (11–35)	9 (4–17)	<0.01
ICU-level care	15,808 (40.1)	3,703 (70.0)	12,105 (35.5)	<0.01
IMV receipt	11,076 (28.1)	3,407 (64.4)	7,669 (22.5)	<0.01
In-hospital death	6,758 (17.1)	2,566 (48.5)	4,192 (12.3)	<0.01

*Values are no. (%) patients except as indicated. ICU, intensive care unit; IMV, invasive mechanical ventilation; NA, not applicable; NH, not Hispanic or Latino. †p values were calculated by using χ^2^ tests for categorical variables or Wilcoxon rank-sum tests for continuous variables, comparing COVID-19–associated and non-COVID-19–associated hospitalizations for fungal infections. Given the heterogeneous nature of fungal infections and their associated risk factors, underlying medical conditions were not analyzed. ‡Sex was unknown for 10 hospitalizations (1 COVID-19–associated, 9 non-COVID-19–associated). Those data were excluded from the χ^2^ test calculation.

Longer hospital stays, higher ICU admission rates, more IMV receipts, and more deaths were generally observed for hospitalizations caused by COVID-19–associated fungal infections than for non–COVID-19–associated fungal infections, regardless of the specific fungal pathogens involved (Appendix Tables 2, 3). COVID-19–associated fungal hospitalizations with the highest percentages of deaths involved aspergillosis (57.6%), invasive candidiasis (55.4%), mucormycosis (44.7%), and unspecified mycoses (59.0%).

## Conclusions

Analysis of a large US healthcare services database indicated that hospitalization rates involving fungal infections increased significantly during 2019–2021, primarily driven by hospitalizations of patients with COVID-19–associated fungal infections. During 2020–2021, a total of 13.4% of fungal hospitalizations were COVID-19–associated, and COVID-19–associated fungal infections were associated with ≈2-fold increase in ICU admission rates and ≈4-fold increase in in-hospital death rates compared with non–COVID-19–associated fungal hospitalizations. Consistent with national mortality data, hospitalizations of patients with COVID-19–associated (compared with non–COVID-19–associated) fungal infections most often involved invasive candidiasis and aspergillosis and disproportionately occurred among non-White male patients in the western United States (*2*). Racial or ethnic disparities observed for fungal infection–associated hospitalization rates might relate to longstanding inequities in social health determinants, such as lack of access to medical care or occupational exposures, and prevalence of underlying conditions, such as diabetes, that might increase fungal and COVID-19 infection risk among certain minority groups (*2*,*7*–*9*). Also consistent with national mortality data, hospitalization rates for COVID-19–associated aspergillosis and mucormycosis increased from 2020 to 2021 (*2*), likely reflecting a greater burden of COVID-19 during 2021 than 2020 (https://gis.cdc.gov/grasp/covidnet/covid19_5.html), increased clinician awareness and testing for COVID-19–associated mold infections (*10*,*11*), and increased use of corticosteroids for COVID-19 treatment, a major risk factor for aspergillosis and mucormycosis (*4*). Our findings emphasize the importance of maintaining a high index of clinical suspicion for fungal infections in patients at high risk, including those with COVID-19, and the need for increased fungal disease surveillance to detect and evaluate emerging trends.

The first limitation of our study is that, although ICD-10-CM codes for COVID-19 correlate well with SARS-CoV-2 test results in PHD-SR data (*12*), fungal ICD-10-CM codes might be associated with underreporting, misclassification, and nonspecific coding of pathogenic fungi, particularly those causing candidemia and invasive mold disease (*13*–*15*). Second, PHD-SR data are broadly representative of US hospitals, and hospital types remained relatively consistent during the analytic period. However, data might overrepresent certain regions of the country, particularly the South, and participating hospitals can vary over time. Finally, we suspect that most COVID-19–associated fungal infections were secondary complications of COVID-19 because of the natural history of fungal disease in patients with respiratory infections (*3*), but we could not verify this supposition by using PHD-SR data.

Our analysis underscores the substantial burden of patient hospitalizations with fungal infections in the United States and indicates that increased hospitalizations involving fungal infections occurred during the COVID-19 pandemic. As the COVID-19 pandemic evolves, and to increase preparedness for future infectious diseases outbreaks, comprehensive public health surveillance for fungal diseases is needed to characterize disease epidemiology and guide efforts to prevent illness and death.

Appendix Additional information for increased hospitalizations involving fungal infections during COVID-19 pandemic, United States, January 2020–December 2021.
