# Supplementary material for: Increased Hospitalizations Involving Fungal Infections during COVID-19 Pandemic, United States, January 2020–December 2021
Source: Emerg Infect Dis. 2023 Jul;29(7):1433–7. doi: 10.3201/eid2907.221771 (PMC10310397; doi:10.3201/eid2907.221771)
Supplement: Appendix — Additional information for increased hospitalizations involving fungal infections during COVID-19 pandemic, United States, January 2020–December 2021. [file 22-1771-Techapp-s1.pdf]

Article DOI: https://doi.org/10.3201/eid2907.221771
EID cannot ensure accessibility for supplementary materials supplied by authors. Readers who have difficulty accessing supplementary content should contact the authors for assistance.

Increased Hospitalizations Involving Fungal Infections during COVID-19 Pandemic, United States, January 2020–December 2021

Appendix

Appendix Table 1. International Classification of Diseases, Tenth Revision, Clinical Modification (ICD-10-CM) discharge diagnosis codes used to identify hospitalizations for fungal infections and COVID-19

Disease	Code, description
Invasive candidiasis	B37.5, candidal meningitis
	B37.6, candidal endocarditis
	B37.7, candidal sepsis
Coccidioidomycosis	B38.0, acute pulmonary coccidioidomycosis
	B38.1, chronic pulmonary coccidioidomycosis
	B38.2, pulmonary coccidioidomycosis, unspecified
	B38.3, cutaneous coccidioidomycosis
	B38.4, coccidioidomycosis meningitis
	B38.7, disseminated coccidioidomycosis
	B38.8, other forms of coccidioidomycosis
	B38.81, prostatic coccidioidomycosis
	B38.89, other forms of coccidioidomycosis
	B38.9, coccidioidomycosis, unspecified
Histoplasmosis	B39.0, acute pulmonary histoplasmosis capsulati
	B39.1, chronic pulmonary histoplasmosis capsulati
	B39.2, pulmonary histoplasmosis capsulati, unspecified
	B39.3, disseminated histoplasmosis capsulati
	B39.4, histoplasmosis capsulati, unspecified
	B39.5, histoplasmosis duboisii
	B39.9, histoplasmosis, unspecified
Blastomycosis	B40.0, acute pulmonary blastomycosis
	B40.1, chronic pulmonary blastomycosis
	B40.2, pulmonary blastomycosis, unspecified
	B40.3, cutaneous blastomycosis
	B40.7, disseminated blastomycosis
	B40.8, other forms of blastomycosis
	B40.81, blastomycotic meningoencephalitis
	B40.89, other forms of blastomycosis
	B40.9, blastomycosis, unspecified
Aspergillosis	B44.0, invasive pulmonary aspergillosis
	B44.1, other pulmonary aspergillosis
	B44.2, tonsillar aspergillosis
	B44.7, disseminated aspergillosis
	B44.8, other forms of aspergillosis
	B44.81, allergic bronchopulmonary aspergillosis
	B44.89, other forms of aspergillosis
	B44.9, aspergillosis, unspecified
Cryptococcosis	B45.0, pulmonary cryptococcosis
	B45.1, cerebral cryptococcosis
	B45.2, cutaneous cryptococcosis
	B45.3, osseous cryptococcosis
	B45.7, disseminated cryptococcosis
	B45.8, other forms of cryptococcosis

Disease	Code, description
	B45.9, cryptococcosis, unspecified
Mucormycosis	B46.0, pulmonary mucormycosis<br>B46.1, rhinocerebral mucormycosis<br>B46.2, gastrointestinal mucormycosis<br>B46.3, cutaneous mucormycosis<br>B46.4, disseminated mucormycosis<br>B46.5, mucormycosis, unspecified<br>B46.8, other zygomycoses<br>B46.9, zygomycosis, unspecified
Other specified mycoses	B41.0, pulmonary paracoccidioidomycosis<br>B41.7, disseminated paracoccidioidomycosis<br>B41.8, other forms of paracoccidioidomycosis<br>B41.9, paracoccidioidomycosis, unspecified<br>B42.0, pulmonary sporotrichosis<br>B42.1, lymphocutaneous sporotrichosis<br>B42.7, disseminated sporotrichosis<br>B42.8, other forms of sporotrichosis<br>B42.81, cerebral sporotrichosis<br>B42.82, sporotrichosis arthritis<br>B42.89, other forms of sporotrichosis<br>B42.9, sporotrichosis, unspecified<br>B43.0, cutaneous chromomycosis<br>B43.1, pheomycotic brain abscess<br>B43.2, subcutaneous pheomycotic abscess and cyst<br>B43.8, other forms of chromomycosis<br>B43.9, chromomycosis, unspecified<br>B48.0, lobomycosis<br>B48.1, rhinosporidiosis<br>B48.2, allescheriasis<br>B48.3, geotrichosis<br>B48.4, penicillosis<br>B48.8, other specified mycoses
Unspecified mycosis	B49, unspecified mycosis
Pneumocystis pneumonia	B59, pneumocystosis
COVID-19	U07.1, COVID-19, virus identified (April 2020–December 2021)<br>B97.29*, other coronavirus as the cause of diseases classified elsewhere (February 2020–April 2020)

*This code for COVID-19 was recommended from March 1, 2020–April 30, 2020 (before the April 2020 release of U07.1) (https://www.cdc.gov/nchs/data/icd/Announcement-New-ICD-code-for-coronavirus-3-18-2020.pdf; https://www.cdc.gov/nchs/data/icd/ICD-10-CM-Official-Coding-Gudance-Interim-Advice-coronavirus-feb-20-2020.pdf).

Appendix Table 2. Demographic features, healthcare utilization, and outcomes for patients hospitalized with COVID-19–associated fungal infections, stratified by fungal pathogen, United States, 2020–2021*

	Cause of infection									
Characteristic	*Candida*	*Aspergillus*	*Coccidioides*	*Pneumocystis*	*Cryptococcus*	*Histoplasma*	Mucorales	*Blastomyces*	Other specified	Unspecified
Total no.	1,135	1,471	723	235	138	139	94	41	221	1,286
Male sex†	663 (58.4)	867 (58.9)	439 (60.7)	164 (69.8)	86 (62.3)	78 (56.1)	72 (76.6)	25 (61.0)	119 (53.8)	767 (59.6)
Median age	64 (54–73)	64 (55–72)	59 (45–71)	58 (47–67)	61 (52–69)	59 (50–70)	55 (44–67)	62 (45–73)	64 (52–74)	64 (54–73)
Age groups, y										
<1	NA	NA	0	0	0	0	0	0	0	0
1–4	NA	0	0	0	0	0	0	0	0	NA
5–14	NA	NA	0	0	0	0	0	0	NA	0
15–24	6 (0.5)	14 (1.0)	19 (2.6)	NA	5 (3.6)	NA	NA	0	NA	8 (0.6)
25–34	34 (3.0)	32 (2.2)	62 (8.6)	16 (6.8)	6 (4.3)	10 (7.2)	8 (8.5)	5 (12.2)	6 (2.7)	46 (3.6)
35–44	92 (8.1)	82 (5.6)	87 (12.0)	26 (11.1)	11 (8.0)	9 (6.5)	13 (13.8)	5 (12.2)	22 (10.0)	88 (6.8)
45–54	147 (13.0)	228 (15.5)	138 (19.1)	56 (23.8)	19 (13.8)	25 (18.0)	22 (23.4)	6 (14.6)	26 (11.8)	179 (13.9)
55–64	293 (25.8)	403 (27.4)	131 (18.1)	63 (26.8)	44 (31.9)	44 (31.7)	21 (22.3)	7 (17.1)	54 (24.4)	327 (25.4)
65–74	317 (27.9)	436 (29.6)	150 (20.7)	40 (17.0)	37 (26.8)	28 (20.1)	19 (20.2)	9 (22.0)	57 (25.8)	362 (28.1)
75–84	189 (16.7)	242 (16.5)	107 (14.8)	27 (11.5)	14 (10.1)	14 (10.1)	8 (8.5)	5 (12.2)	42 (19.0)	219 (17.0)
≥85	52 (4.6)	32 (2.2)	29 (4.0)	NA	NA	5 (3.6)	0	NA	10 (4.5)	55 (4.3)
Race/ethnicity										
White, NH	426 (37.5)	739 (50.2)	137 (18.9)	81 (34.5)	34 (24.6)	81 (58.3)	31 (33.0)	25 (61.0)	107 (48.4)	547 (42.5)
Black, NH	223 (19.6)	161 (10.9)	37 (5.1)	55 (23.4)	27 (19.6)	17 (12.2)	16 (17.0)	10 (24.4)	33 (14.9)	223 (17.3)
Hispanic or Latino	254 (22.4)	258 (17.5)	138 (19.1)	38 (16.2)	40 (29.0)	14 (10.1)	27 (28.7)	NA	40 (18.1)	230 (17.9)
Asian, NH	19 (1.7)	38 (2.6)	NA	NA	5 (3.6)	NA	NA	0	6 (2.7)	33 (2.6)
Other	42 (3.7)	57 (3.9)	14 (1.9)	10 (4.3)	8 (5.8)	14 (10.1)	NA	NA	9 (4.1)	57 (4.4)
Unknown	171 (15.1)	218 (14.8)	394 (54.5)	49 (20.9)	24 (17.4)	11 (7.9)	14 (14.9)	NA	26 (11.8)	196 (15.2)
US census region										
South	592 (52.2)	614 (41.7)	18 (2.5)	83 (35.3)	64 (46.4)	61 (43.9)	43 (45.7)	16 (39.0)	107 (48.4)	639 (49.7)
West	197 (17.4)	365 (24.8)	697 (96.4)	30 (12.8)	25 (18.1)	7 (5.0)	28 (29.8)	NA	42 (19.0)	190 (14.8)
Midwest	186 (16.4)	322 (21.9)	6 (0.8)	74 (31.5)	23 (16.7)	67 (48.2)	14 (14.9)	22 (53.7)	47 (21.3)	219 (17.0)
Northeast	160 (14.1)	170 (11.6)	NA	48 (20.4)	26 (18.8)	NA	9 (9.6)	NA	25 (11.3)	238 (18.5)
Urbanicity										
Urban	1,050 (92.5)	1,321 (89.8)	710 (98.2)	214 (91.1)	125 (90.6)	114 (82.0)	86 (91.5)	30 (73.2)	185 (83.7)	1,148 (89.3)
Rural	85 (7.5)	150 (10.2)	13 (1.8)	21 (8.9)	13 (9.4)	25 (18.0)	8 (8.5)	11 (26.8)	36 (16.3)	138 (10.7)
Healthcare utilization and outcomes										
Length of stay, d	29 (17, 45)	23 (14, 35)	8 (5, 15)	18 (9, 33)	19.5 (8, 31)	10 (4, 24)	18 (9, 30)	11 (5, 26)	21 (13, 33)	25 (15, 39)
ICU-level care	932 (82.1)	1,181 (80.3)	210 (29.0)	146 (62.1)	83 (60.1)	51 (36.7)	66 (70.2)	19 (46.3)	152 (68.8)	1,030 (80.1)
IMV receipt	884 (77.9)	1,115 (75.8)	159 (22.0)	115 (48.9)	71 (51.4)	37 (26.6)	54 (57.4)	17 (41.5)	131 (59.3)	977 (76.0)
In-hospital death	629 (55.4)	848 (57.6)	135 (18.7)	96 (40.9)	53 (38.4)	30 (21.6)	42 (44.7)	10 (24.4)	82 (37.1)	759 (59.0)

*Values are median (interquartile range) for continuous variables (median age) and number (%) for categorical variables. Cells containing <5 hospitalizations are not shown. ICU, intensive care unit; IMV, invasive mechanical ventilation; NA, not applicable; NH, not Hispanic or Latino.
†Sex was unknown for 1 hospitalized patient.

Appendix Table 3. Demographic features and outcomes for patients hospitalized with non-COVID-19-associated fungal infection during the COVID-19 pandemic, stratified by pathogen, United States, 2020–2021*

	Cause of infection									
Characteristics	*Candida*	*Aspergillus*	*Coccidioides*	*Pneumocystis*	*Histoplasma*	*Cryptococcus*	*Blastomyces*	Mucorales	Other specified	Unspecified
Total no.	7,154	5,777	5,555	3,483	2,247	1,884	502	475	2,079	6,313
Male sex†	3,702 (51.7)	3,288 (56.9)	3,355 (60.4)	1,184 (34.0)	1,328 (59.1)	1,310 (69.5)	347 (69.1)	298 (62.7)	1,091 (52.5)	3,377 (53.5)
Median age	64 (50–74)	64 (53–73)	57 (42–70)	52 (38–63)	58 (41–69)	55 (42–66)	60 (43–70)	55 (42–65)	63 (49–74)	63 (49–73)
Age group, y										
<1	34 (0.5)	14 (0.2)	NA	8 (0.2)	NA	0	0	NA	11 (0.5)	41 (0.6)
1–4	33 (0.5)	19 (0.3)	NA	10 (0.3)	16 (0.7)	NA	NA	NA	18 (0.9)	33 (0.5)
5–14	61 (0.9)	72 (1.2)	48 (0.9)	18 (0.5)	30 (1.3)	7 (0.4)	NA	6 (1.3)	37 (1.8)	59 (0.9)
15–24	133 (1.9)	186 (3.2)	298 (5.4)	71 (2.0)	91 (4.0)	54 (2.9)	22 (4.4)	29 (6.1)	80 (3.8)	131 (2.1)
25–34	469 (6.6)	248 (4.3)	565 (10.2)	579 (16.6)	224 (10.0)	199 (10.6)	46 (9.2)	32 (6.7)	105 (5.1)	419 (6.6)
35–44	622 (8.7)	386 (6.7)	696 (12.5)	573 (16.5)	283 (12.6)	290 (15.4)	63 (12.5)	73 (15.4)	146 (7.0)	576 (9.1)
45–54	880 (12.3)	625 (10.8)	923 (16.6)	675 (19.4)	325 (14.5)	359 (19.1)	72 (14.3)	92 (19.4)	256 (12.3)	829 (13.1)
55–64	1,511 (21.1)	1,416 (24.5)	1,075 (19.4)	751 (21.6)	476 (21.2)	448 (23.8)	111 (22.1)	116 (24.4)	455 (21.9)	1,341 (21.2)
65–74	1,761 (24.6)	1,610 (27.9)	1,035 (18.6)	476 (13.7)	479 (21.3)	347 (18.4)	97 (19.3)	84 (17.7)	496 (23.9)	1,474 (23.3)
75–84	1,191 (16.6)	990 (17.1)	700 (12.6)	258 (7.4)	267 (11.9)	152 (8.1)	65 (12.9)	32 (6.7)	319 (15.3)	1,016 (16.1)
≥85	459 (6.4)	211 (3.7)	212 (3.8)	64 (1.8)	55 (2.4)	27 (1.4)	22 (4.4)	8 (1.7)	156 (7.5)	394 (6.2)
Race/ethnicity										
White, NH	3,845 (53.7)	3,251 (56.3)	1,130 (20.3)	1,322 (38.0)	1,394 (62.0)	716 (38.0)	296 (59.0)	211 (44.4)	1,212 (58.3)	3,481 (55.1)
Black, NH	1,227 (17.2)	723 (12.5)	244 (4.4)	979 (28.1)	328 (14.6)	414 (22.0)	81 (16.1)	56 (11.8)	262 (12.6)	1,060 (16.8)
Hispanic or Latino	793 (11.1)	581 (10.1)	761 (13.7)	477 (13.7)	205 (9.1)	389 (20.6)	33 (6.6)	82 (17.3)	232 (11.2)	657 (10.4)
Asian, NH	108 (1.5)	225 (3.9)	102 (1.8)	99 (2.8)	41 (1.8)	70 (3.7)	23 (4.6)	8 (1.7)	27 (1.3)	95 (1.5)
Other	234 (3.3)	190 (3.3)	132 (2.4)	104 (3.0)	115 (5.1)	64 (3.4)	31 (6.2)	16 (3.4)	63 (3.0)	231 (3.7)
Unknown	947 (13.2)	807 (14.0)	3,186 (57.4)	502 (14.4)	164 (7.3)	231 (12.3)	38 (7.6)	102 (21.5)	283 (13.6)	789 (12.5)
US census region										
South	3,662 (51.2)	2,407 (41.7)	295 (5.3)	1,655 (47.5)	1,128 (50.2)	1,061 (56.3)	185 (36.9)	201 (42.3)	1,048 (50.4)	3,124 (49.5)
West	1,041 (14.6)	1,270 (22.0)	5,144 (92.6)	522 (15.0)	102 (4.5)	262 (13.9)	8 (1.6)	165 (34.7)	281 (13.5)	831 (13.2)
Midwest	1,432 (20.0)	1,266 (21.9)	91 (1.6)	690 (19.8)	915 (40.7)	281 (14.9)	283 (56.4)	72 (15.2)	454 (21.8)	1,224 (19.4)
Northeast	1,019 (14.2)	834 (14.4)	25 (0.5)	616 (17.7)	102 (4.5)	280 (14.9)	26 (5.2)	37 (7.8)	296 (14.2)	1,134 (18)
Urbanicity										
Urban	6,438 (90.0)	5,260 (91.1)	5,399 (97.2)	3,236 (92.9)	2,024 (90.1)	1,695 (90.0)	430 (85.7)	439 (92.4)	1,825 (87.8)	5,612 (88.9)
Rural	716 (10.0)	517 (8.9)	156 (2.8)	247 (7.1)	223 (9.9)	189 (10.0)	72 (14.3)	36 (7.6)	254 (12.2)	701 (11.1)
Healthcare utilization and outcomes										
Length of stay, d	13 (7, 24)	8 (4, 16)	5 (3, 10)	9 (5,16)	6 (3, 12)	11 (5, 20)	6 (3, 13)	11 (4, 24)	7 (4, 15)	11 (6, 21)
ICU-level care	3,642 (50.9)	1,953 (33.8)	1,172 (21.1)	1,221 (35.1)	606 (27.0)	532 (28.2)	152 (30.3)	200 (42.1)	581 (27.9)	2,689 (42.6)
IMV receipt	2,429 (34.0)	1318 (22.8)	526 (9.5)	805 (23.1)	326 (14.5)	315 (16.7)	84 (16.7)	114 (24.0)	342 (16.5)	1,839 (29.1)
In-hospital death	1,267 (17.7)	761 (13.2)	248 (4.5)	565 (16.2)	162 (7.2)	188 (10.0)	45 (9.0)	71 (14.9)	169 (8.1)	976 (15.5)

*Values are median (interquartile range) for continuous variables (median age) and number (%) for categorical variables. Cells containing <5 hospitalizations are not shown. ICU, intensive care unit; IMV, invasive mechanical ventilation; NA, not applicable; NH, not Hispanic or Latino.
†Sex was unknown for 9 hospitalized patients.
